# Complete Genome Sequence of a Toxic and Bioactive Exopolysaccharide-Bearing Bacterium, *Sulfitobacter* sp. Strain AM1-D1

**DOI:** 10.1128/MRA.00232-20

**Published:** 2020-04-16

**Authors:** Xi Yang, Zhi-Wei Jiang, Zhuang Chen, Jun Dai, Lei Wang, Xiao-Ling Zhang, Qiao Yang

**Affiliations:** a Agro-Biological Gene Research Center, Guangdong Academy of Agricultural Sciences, Guangzhou, People’s Republic of China; b ABI Group, Zhejiang Ocean University, Zhoushan, People’s Republic of China; c Phycosphere Microbiome Project Consortium, Shanghai, People’s Republic of China; Georgia Institute of Technology

## Abstract

*Sulfitobacter* sp. strain AM1-D1, a toxic bacterium of the family *Rhodobacteraceae*, was isolated from the cultivable phycosphere microbiota of marine toxigenic dinoflagellate Alexandrium minutum amtk4. The complete 4.69-Mb genome comprises one single circular chromosome and five circular plasmids. It has 4,559 coding genes, including those for biosynthesis or degradation of saxitoxin and bioactive exopolysaccharides.

## ANNOUNCEMENT

The phycosphere is a microscopic niche harboring interkingdom exchanges of diverse nutrients, infochemicals, and transferable genetic elements through complex alga-bacterium interactions (ABI) (1). Phycosphere microbiota (PM) inhabiting this microenvironment as a whole bacterial community has been reported in numbers of phytoplanktons in aquatic environments (2, 3). Here, we report the complete genome sequence of *Sulfitobacter* sp. strain AM1-D1, which was isolated from the cultivable PM of marine toxigenic dinoflagellate Alexandrium minutum amtk4 that produces paralytic shellfish poisoning toxins (PSTs) (4). This genomic information will enable improved mapping of bacterial roles during these dynamic interactions.

The bacterial isolation and extraction of genomic DNA were performed according to our previously described protocols (5). The complete genome sequence of *Sulfitobacter* sp. strain AM1-D1 was acquired by PacBio RS II single-molecule real time (SMRT) technology performed at Majorbio (Shanghai, China). For PacBio sequencing, genomic DNA was sheared to a standard 10-kb size using g-TUBE (Covaris) and converted into a proprietary SMRTbell library format using the RS DNA template preparation kit (Pacific Biosciences, CA) according to the manufacturer’s instructions. Default parameters were used in this study for all software unless otherwise specified. A quality check of raw sequences was performed using FastQC (http://www.bioinformatics.babraham.ac.uk/projects/fastqc). The genome sequence was *de novo* assembled from PacBio reads (186,958 reads; average length, 5,769 bp; coverage, 230×) into six contigs using SOAPdenovo2 v2.04 with the Fast Alignment and CONsensus (FALCON) v0.2.1 tools and circularized with Circos v0.64 (http://circos.ca). The 4.69-Mb genome of *Sulfitobacter* sp. strain AM1-D1 comprises one single circular chromosome (3,840,209 bp) and five circular plasmids (185,176 bp, 95,272 bp, 15,811 bp, 205,668 bp, and 348,874 bp). The G+C contents of these components are 64.9%, 65.6%, 60.3%, 58.7%, 63.7%, and 61.6%, respectively. The genome sequence was annotated by NCBI Prokaryotic Genome Annotation Pipeline (PGAP) v1.2.1 (6). It harbors 43 tRNAs and 3 rRNAs. Of the 4,559 predicted coding genes, the key *sxtA* genes for starting the biosynthesis of saxitoxin (4) and those for the biosynthesis or degradation of cross-kingdom signals, namely, indole-3-acetic acid (IAA) (2) and bioactive exopolysaccharides (EPS) as promising natural bioflocculants (7), are present in the bacterial chromosome.

### Data availability.

The annotated complete genome sequence of *Sulfitobacter* sp. strain AM1-D1 was deposited in DDBJ/ENA/GenBank under the accession numbers CP018076, CP018077, CP018078, CP018079, CP018080, and CP018081 for the single circular chromosome and five circular plasmids, respectively. The raw reads are available under SRA accession number SRR11216889.
